# Chiral cerium doped nanoreactor-mediated bladder carcinoma reinforced ablation *via* fluorescence imaging navigated surgery along with photothermal strengthened ROS storm

**DOI:** 10.1016/j.mtbio.2026.103746

**Published:** 2026-10-09

**Authors:** Guangwei Zheng, Ao Wang, Yiqing Du, Caipeng Qin, Yuxuan Song, Jilin Wu, Xing Luo, Xiaolong Liu, Peiyuan Wang, Tao Xu

**Affiliations:** a Department of Urology, Peking University People's Hospital, Beijing, 100044, PR China; b The United Innovation of Mengchao Hepatobiliary Technology Key Laboratory of Fujian Province, Mengchao Hepatobiliary Hospital of Fujian Medical University, Fuzhou, 350025, PR China; c Fujian Institute of Research on the Structure of Matter, Chinese Academy of Sciences, Fuzhou, 350002, PR China

**Keywords:** Chiral nanostructure, Fluorescence enhancement, Higher endocytosis, Strong catalysis, Tumor suppression

## Abstract

Bladder cancer is regarded as one of the most prevalent malignant neoplasms of the urinary tract. However, the treatment of bladder cancer still presents unsatisfactory therapeutic outcomes, especially for advanced patients. Chiral nanomaterials hold significant prospect for elevating clinical therapeutic effect, thanks to their unparalleled chiral selectivity in biological-systems. In this work, we have engineered chiral a cerium oxide (CeO_2_) doped mesoporous silica with, a photothermal and fluorescence contrast agent, indocyanine green loading (cSiCe&ICG). On account of the unique chiral nanostructure, this fascinating nanoreactor manifested effective enrichment in bladder tumor tissues and might be activated to mediate robust reactive oxygen species (ROS) under the weak acid and high H_2_O_2_ microenvironment of the tumor regions. Owing to the accurate tumor targeting and fluorescence intensification, increased tumor-to-background ratio (TBR) had been achieved for accurately discriminating tumor tissues from surrounding normal tissue. With the navigation of fluorescence imaging at second near-infrared region (NIR II, 1000-1700 nm), tumors could be completely removed. In particularly, with the aid of 808 nm light irradiation, the chiral Ce-based nanoreactor exhibited a high catalytic efficiency, by integrating photothermal that further mediating ROS production *via* Ce ions for bladder tumor cell eradication. These findings underscored the prospect of chiral nanoreactor for efficacious fluorescence bioimaging for guiding tumor surgery and synergistic tumor eradiation especially with the assistance of NIR light activation.

## Graphical abstract

**Figure:**
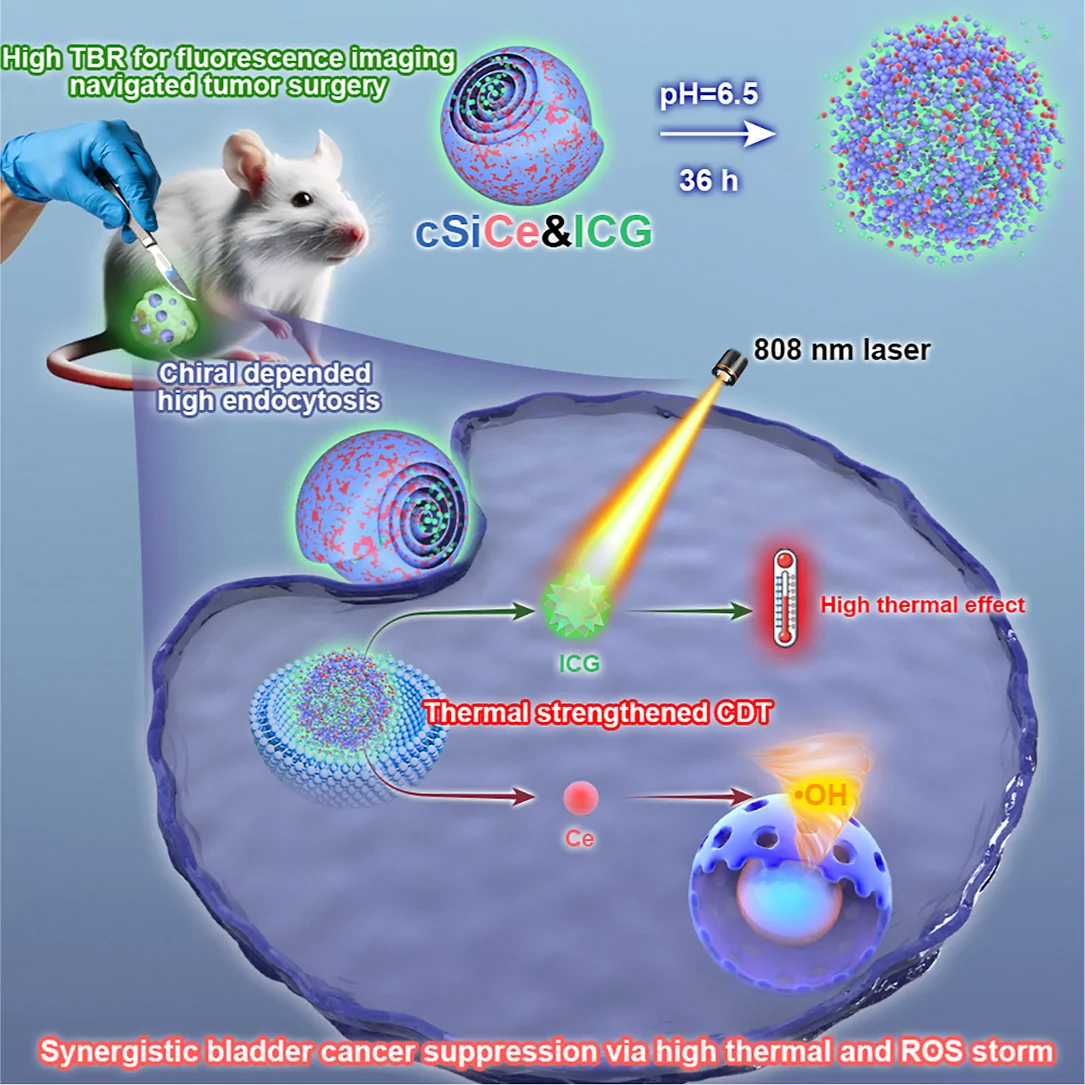

## 1 Introduction

Bladder cancer (BC) ranks among the most common genitourinary cancers, with over 600,000 new cases reported globally every year. Up to 75% of all bladder cancer cases are categorized as non-muscle invasive [1], [2], [3]. The standard approach for treating non-muscle invasive bladder cancer typically involves transurethral resection of the tumor combined with postoperative instillation therapy [4], [5]. Approximately 63-98% of patients receive surgical intervention for bladder cancer [6], [7]. To minimize invasiveness, it is crucial to obtain clear anatomical visualization prior to surgery. Real-time imaging of the surgical area provides essential anatomical information during the procedure [8], [9], [10]. Advances in optical imaging technologies have significantly improved the accuracy of intraoperative solid tumor detection. In recent years, bioimaging agents that have received clinical approval, such as indocyanine green (ICG) and methylene-blue (MB), which can be activated by near-infrared (NIR) lasers, have shown potential in identifying bladder cancer, locating lymph nodes, and detecting metastatic sites [11], [12], [13]. Importantly, ICG demonstrates fluorescence in both the first near-infrared (NIR) window (700-900 nm, NIR I) and the second near-infrared window (1000-1700 nm, NIR II) [14], [15], [16]. The characteristic enhances the biological imaging efficiency. When expose to an 808 nm light source, ICG emits light in the NIR II, which is able to be detected using specialized InGaAs cameras. These biological images offer an superior tumor-to-background ratio (TBR) compared to conventional NIR I techniques [17], [18]. This enhancement is particularly advantageous for more accurate imaging of deeper tissues. Unfortunately, the clinical use of ICG is constrained by its rapid clearance from the bloodstream, poor retention and enrichment in tumor tissues, and its strong propensity to associate with serum albumin [19], [20]. These drawbacks compromise the overall performance of ICG in biomedical applications. Consequently, designing next-generation NIR-II nanoprobes offers promising avenues for constructing targeted nanocarriers that can enhance bladder cancer imaging and, in turn, improve clinical outcomes.

Chiral molecules are those that exist in two forms that are mirror images of each other, and their chirality plays a crucial role in biological systems [21], [22], [23]. In living organisms, chiral components typically occur in only one enantiomeric form. For example, nucleotides and sugars predominantly exist in the D-configuration, whereas amino acids and phospholipids are mainly in the L-form [24], [25], [26]. These chiral substances are essential for various physiological processes, including metabolism and cellular fate [27], [28], [29]. Chiral drugs can lead to different or even opposing biological effects; for instance, L-Dopa is used in the treatment of Parkinson's disease, while ibuprofen serves as an anti-inflammatory and pain-relieving agent [30]. Recently, research on chirality has extended beyond organic compounds to include inorganic nanoparticles [31], [32]. Due to their unique photochemical properties, chiral nanoparticles have found applications in areas such as regulating biological behaviors, chiral sensing, separation of enantiomers, and chiral catalysis [33], [34]. Nevertheless, the study of ROS generation induced by chiral catalysis in tumor cells remains largely unexplored [35], [36]. In recent years, chemodynamic therapy (CDT), which employs Fe-based nanoagents to catalyze H_2_O_2_ decomposition through the Fenton reaction and generate •OH radicals, has been regarded as a promising approach for carcinoma treatment [37], [38]. The acidic environment within tumors and the Fenton-like reactions mediated by nanoreactors have also drawn significant attention. Among these, cerium-based nanomaterials stand out due to their ability to switch between Ce^4+^ and Ce^3+^ oxidation states [39], [40]. Cerium oxide systems can decompose H_2_O_2_ and deplete glutathione (GSH) through redox reactions, thereby inducing substantial oxidative stress in the cytoplasm of tumor cells [41], [42]. Hence, developing CeO_2_ nanoparticles with chiral catalytic activity is expected to improve catalytic performance in tumor cells, particularly when combined with the photothermal effect of ICG [43], [44], leading to a significant increase in ROS production.

Taking into account the above challenges, as illustrated in Fig. 1, we proposed a lamellar micelle spiral self-assembly method within a biphasic stratification system to fabricate multi-layered mesoporous silica nanocarriers doped with CeO_2_, exhibiting distinct chirality (cSiCe). Latterly, as a fluorescent dye suitable for NIR II imaging and photothermal therapy (PTT), ICG was encapsulated within the chiral channels to form cSiCe&ICG. The distinctive chiral structure of cSiCe&ICG demonstrates sustained fluorescence enhancement in the NIR II region, excellent cellular uptake efficiency, and complete removal of BC tumors under NIR II fluorescence-guided bioimaging with a satisfied TBR. Moreover, the chiral catalytic efficiency of CDT from CeO_2_ was effectively achieved, enhancing the formation of a ROS storm by increasing the local temperature *via* ICG under NIR laser irradiation. Based on the complete resection of the tumor and the synergistic antitumor effects, this strategy demonstrated effective suppression of BC. To the best of our knowledge, this represented the first facile fabrication of a chiral CeO_2_ nanoreactor for precise and synergistic treatment of malignant tumors.

**Fig. 1:**
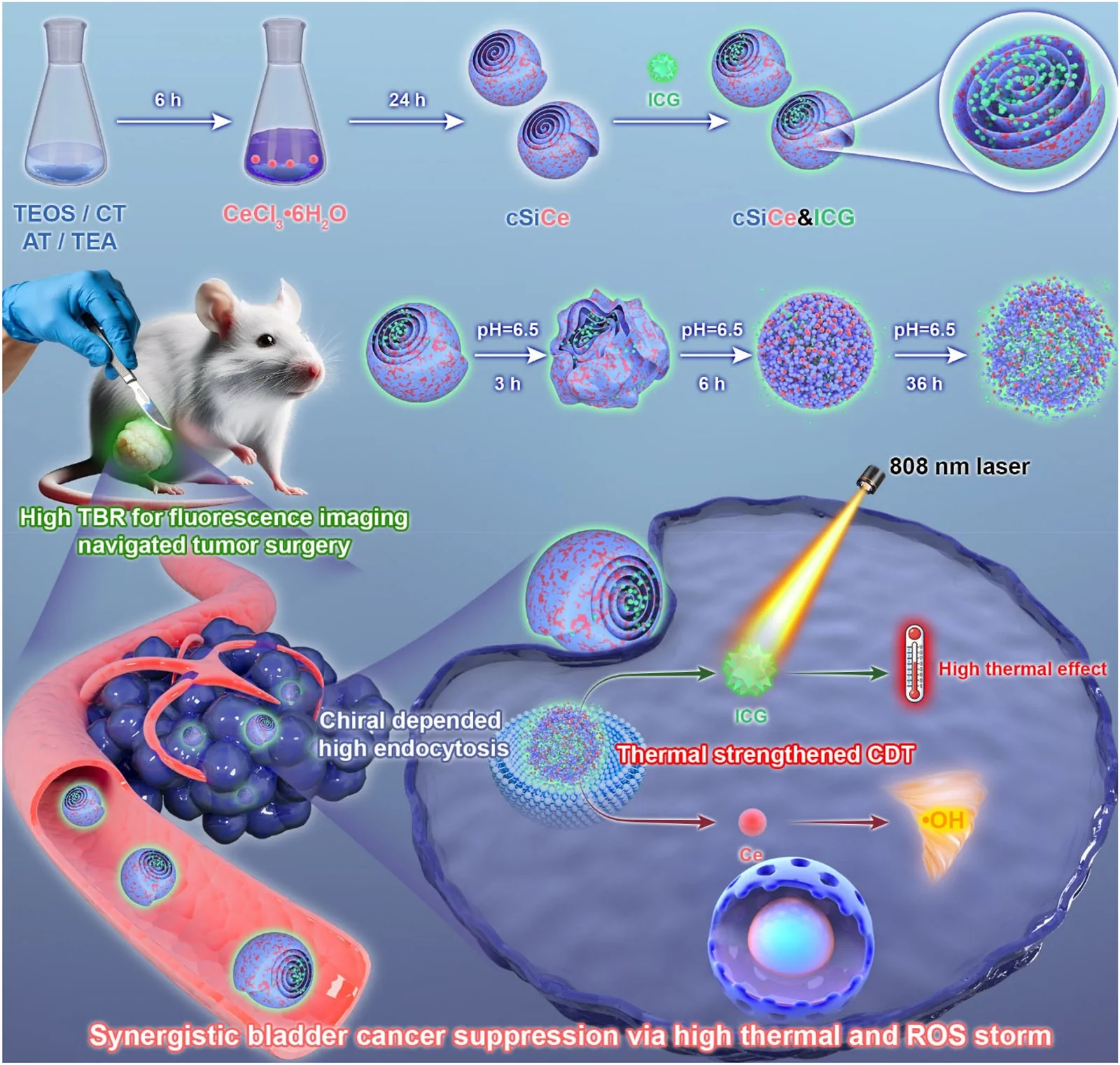
Scheme illustration of tumor microenvironment responsive chiral cSiCe&ICG nanoreactor construction for bladder cancer surgery under NIR II fluorescence imaging with high TBR and synergistic suppression *via* high thermal and ROS storm.

## 2 Experimental

### 2.1 Preparation of cSiCe

cSiCe was synthesized using a general assembly method based on the lamellar micelle spiral self-assembly approach. Specifically, 1.5 g of hexadecyltrimethylammonium p-toluenesulfonate (CTAT) was dissolved in 60 mL of water, and then 0.72 mL of 25% triethanolamine (TEA) was added. The aqueous solution was gently stirred at 60°C, after which 16 mL of cyclohexane and 4 mL of tetraethyl orthosilicate (TEOS) were carefully introduced on top of the aqueous layer. The reaction was carried out for 6 h, followed by the addition of 45 mg of CeCl_3_•6H_2_O into the aqueous phase. After stirring for an additional 16 h, cSiCe nanoparticles were successfully formed. The resulting product was washed three times with ethanol and three times with water. After washing, the material was dispersed in water for subsequent applications. In parallel, cSiCe samples were also prepared at different reaction times: 6 h, 12 h, and 24 h.

### 2.2 Preparation of cSiCe&ICG

The final cSiCe&ICG composite was fabricated through the following procedure: Initially, 1 mg of ICG powder was dissolved in 10 mL of distilled water. Then, 10 mL of a 1 mg/mL cSiCe aqueous solution was mixed with the ICG solution. The mixture was vigorously stirred at room temperature overnight to allow efficient adsorption of ICG molecules into the mesoporous structure of cSiCe. Following centrifugation, the sample was washed three times with water to remove any unbound ICG. Finally, the cSiCe&ICG nanoplatform was re-suspended in 5 mL of water for further use.

### 2.3 *In vitro* photostability assessment

A total of 200 μL of cSiCe&ICG solution at a concentration of 200 μg/mL was placed into each well of a 96-well plate. As a control, an ICG solution with comparable fluorescence intensity was also added to the same plate. Both samples were continuously exposed to an 808 nm near-infrared (NIR) laser for different time intervals (0-5 min). Immediately after exposure, all samples were observed using a custom-built NIR II bio-imaging system (808 nm laser, power density = 100 mW/cm^2^) equipped with a long-pass filter set at 1000 nm. The corresponding NIR II fluorescence intensities were recorded quantitatively to evaluate the photostability of the chiral nanostructures. Additionally, the same samples were kept under normal laboratory lighting for various durations (1, 2, 4, 6, 8, 12, and 24 h), and their fluorescence intensities were measured using the same imaging setup and testing conditions.

### 2.4 Cellular by bladder carcinoma cells

The internalization of cSiCe&ICG by cells was analyzed using confocal laser scanning microscopy (CLSM). Initially, ICG was incorporated into cSiCe and mSiCe to produce cSiCe&ICG and mSiCe&ICG, respectively. Subsequently, MB49 bladder cancer cells were seeded in 12-well plates at a density of 2 × 10^5^ cells per well in 1 mL of fresh DMEM medium containing 10% fetal bovine serum, 100 U/mL penicillin, and 100 μg/mL streptomycin. The cells were then co-cultured with the nanoparticles for 24 h. Afterward, the cells were separately exposed to 100 μg/mL of cSiCe&ICG. At various time points, including 1, 2, 4, 8, and 12 h post-incubation, the samples were rinsed three times with 1× PBS and fixed with 4% paraformaldehyde. To visualize the nuclei, the cells were stained with 1 μg/mL DAPI for 20 min before being examined under CLSM. In addition, MB49 bladder tumor cells were seeded in 6-well plates at a concentration of 4 × 10^5^ cells per well in 1 mL of fresh DMEM medium (10% FBS, 100 U/mL penicillin, 100 μg/mL streptomycin) and incubated for 24 h. Following treatment with free ICG, 100 μg/mL of cSiCe&ICG, and an equivalent concentration of mSiCe&ICG, the cells were washed three times with 1× PBS after an 8-h incubation period and collected *via* enzymatic digestion. Nuclei were stained with 1 μg/mL DAPI for 20 min, and the uptake capacity was assessed using CLSM.

### 2.5 ROS detection in bladder carcinoma cells

A cytoplasmic investigation was conducted to evaluate the generation of ROS by Fenton-like catalytic reactions mediated by Ce^3+^ from the internalized cSiCe&ICG in bladder cancer cells (MB49) under the observation of CLSM. BC cell was incubated in Dulbecco's Modified Eagle Medium (DMEM) added with fetal bovine serum (FBS) and antibiotics. The corresponded protocol was as carried out: all BC cells were pre-incubated with H_2_O_2_ (100 μM) for about 2 h. Subsequently, the MB49 cells were co-incubated with the cSiCe&ICG nanomedicine. After an 8-h incubation, the sample was irradiated by an 808 nm light for 5 min (1 W/cm^2^, referred to “L”). Other groups including tumor cells treated with ICG alone, cSiCe&ICG alone, and mSiCe&ICG combined with laser exposure. Latterly, ROS content was visualized using a commercial probe, 2′,7′-Dichlorodihydrofluorescein Diacetate (DCFH-DA), known for its green fluorescence emission (excitation at 488 nm and emission at 550 nm). After an additional 30 min of incubation, the cells were examined using a CLSM to evaluate the ROS generation within the tumor cells triggered by the chiral nanoparticles.

### 2.6 Mitochondrial membrane potential staining

The damage of mitochondrial membrane potential (MMP) caused by the oxidation of ROS was apprised after staining by JC-1. 1 × 10^5^ of MB49 carcinoma cells per well, were cultured in 6-well plates for a duration of 24 h. Immediately, the cells were randomly assigned into five distinct experimental groups: (1) the PBS control group, (2) the ICG with low concentration (ICG + L) group, (3) cSiCe&ICG group, (4) mSiCe&ICG + L group, and (5) cSiCe&ICG + L group. Following an 8-h incubation with the nanoparticles, groups designated for laser treatment were irradiated with 808 nm light for a duration of 5 min at a power density of 1 W/cm^2^. The aggregates and monomers of JC-1 were measured for their red and green fluorescence, respectively, with spectral separation (red aggregates: excitation/emission at 530 nm/590 nm and green monomers: excitation/emission at 488 nm/530 nm). Depolarization ratios were then calculated by comparing the differences in fluorescence intensities.

### 2.7 *In vivo* targeting of tumor sites and photothermal generation

BALB/c mice (Six-week-old, female) were obtained from GemPharmatech Co., Ltd. All animal experiments were approved by the Ethics Committee for Laboratory Animal Research at Peking University Health Science Center (2025PHB139-001). Subcutaneous bladder cancer models in mice were created by injecting MB49 cells (50 μL, 1 × 10^6^ cells in 1X PBS) into the muscle of the right hind limb of 6-week-old female BALB/c mice. When the tumor volume reached 200 mm^3^, the mice were administered intravenous injections of ICG, mSiCe&ICG, or cSiCe&ICG, respectively. After injection, a self-made near-infrared II fluorescence bio-imaging system (NIR-OPTICS Series III, equipped with a long-pass filter at 1000 nm) was used to take images at various time points post-injection. After 12 h, key organs and tumors from two groups were surgically removed for *ex vivo* fluorescence imaging.

To evaluate the photothermal effect, PBS and cSiCe&ICG were injected separately into the caudal veins of BALB/c mice. Three h after injection, the tumor tissue was exposed to an 808 nm near-infrared laser (1.5 W/cm^2^) for 5 min. Thermal images were then recorded using a photothermal camera (FOTRIC 225s).

### 2.8 *In vivo* anti-tumor evaluation

When the subcutaneous tumor size reached approximately 100 mm^3^, tumor-bearing mice were randomly divided into five groups (n = 4 per group). The mice received injections into the caudal vein of different treatment agents, including PBS + L, cSiCe + L, mSiCe&ICG + L, and cSi&ICG + L, with each group receiving a silica-based nanoparticle at a dose of 8.0 mg/kg. Twelve h later, all mice were exposed to light illumination (808 nm, 1.5 W/cm^2^, 6 min). Tumor volume, body weight was recorded before injection and every three days during the whole treatment period. The ultimate tumor volume was determined using the formula: length × width^2^/2. After seven days of treatment, all mice were humanely euthanized, and vital organs such as the heart, liver, spleen, lungs, and kidneys, along with the tumor tissues, were collected for histopathological analysis.

### 2.9 Statistical analysis

The data are presented as mean ± standard deviation. Differences between groups were analyzed using one-way analysis of variance (ANOVA). A p-value of *p < 0.05 indicates a statistically significant difference, while **p < 0.01 indicates a highly significant difference among the groups.

## 3 Results and discussion

### 3.1 Fabrication of chiral Ce-doped mesoporous silica

Chiral nanostructured mesoporous silica (cSi) doped with CeO_2_ can be synthesized through a heterogeneous oil/water biphasic stratification reaction system (Fig. 1). The upper oil phase comprises TEOS dissolved in hydrophobic cyclohexane, whereas the lower aqueous phase contains CTAT as the structure-directing template and TEA as the organic catalyst. A multishelled chiral mesostructure is subsequently formed through a one-pot, continuous interfacial growth process. Consistent with previous study [45], our results suggest that the helical self-assembly of lamellar micelles occurs at the oil-water interface. Upon stirring at 300 rpm, CTAT, TEA, and TEOS coassemble into lamellar micelles within the reaction system. The Ce precursor (CeCl_3_•6H_2_O) was added at 0, 6, or 12 h after the addition of TEOS/cyclohexane. TEM images showed that early addition resulted in phase separation, whereas late addition led to a relatively low Ce content (weight ratio of 1:7) (Fig. S1). Therefore, cSiCe prepared by adding the Ce precursor at 6 h (weight ratio of 2:7) was selected for subsequent experiments. Consistent with prior research, our results showed that lamellar micelle spiral self-assembly occurs at the water/oil interface. Under continuous stirring at 300 rpm, lamellar micelles composed of CTAT, TEA, CeO_2_, and TEOS formed within the system. As shown in the transmission electron microscopy (TEM) images in Fig. 2A, the initial stage of cSi revealed small, slightly aggregated particles beginning to assemble (after 6 h). Subsequently, the Ce precursor underwent hydrolysis, leading to nucleation and growth within the assembled structures. As the reaction continues, these small Ce-doped silica nanoparticles gradually experienced spiral self-assembly, forming intermediate multi-layered structures (after 12 h, Fig. 2B). Eventually, well-defined spiral multi-layered silica nanospheres with a fuzzy or hairy appearance, similar to lamellar micelles, were observed (after 24 h, Fig. 2C). Extending the reaction time to 48 h leads to denser interlayers, forming clasp-ring structures, and an increased number of micelles were deposited on the surface (Fig. 2D). High-resolution transmission electron microscopy (HRTEM) and high-angle annular dark-field scanning transmission electron microscopy (HAADF-STEM) observations indicated that the mesoporous silica nanospheres produced after 24 h of stirring are highly uniform, with an average nanoparticle size of approximately 170 nm (Fig. 2E and F). Therefore, we selected the samples obtained after a 24 h reaction for subsequent biological applications. Field-emission scanning electron microscopy (FESEM) images clearly showed residual micelles on the surface of the chiral nanoagent (cSiCe), which create a rough texture that enhances membrane binding and facilitates cellular internalization (Fig. S2). Detailed HRTEM observations and images revealed that the cSiCe nanoparticles exhibit both clockwise (denoted as “CW”, Fig. 2G) and counterclockwise (denoted as “CCW”, Fig. 2H) chirality. As previously reported chiral helical materials, such as mesoporous polydopamine [46], cSiCe also generally existed as mixtures of both helical configurations. Meaningfully, multi-layered chiral hollow structures had a mean interlayer spacing of about 3 nm between adjacent layers, while the outer gaps were larger than ∼10 nm (Fig. 2G and H). Elemental mapping of individual spiral cSiCe structures was presented in Fig. 2I, clearly illustrating the presence of silicon (Si), oxygen (O), and cerium (Ce) across each Ce-based silica shell. The specific surface area of cSiCe was appraised by nitrogen adsorption-desorption measurements to be 251.227 m^2^/g, with an average pore size of 5.08 nm (Fig. S3). These results demonstrate that cSiCe has the potential to serve as a drug carrier for targeted delivery. Furthermore, when ICG dyes were incorporated into the chiral mesopores (cSiCe&ICG), some CeO_2_ nanogranules were found to anchor onto the structure (Fig. 2J). The CeO_2_ nanoenzymes are anchored to the surface of cSi, with some Ce species also incorporated into the silica framework. No Ce ions were detected after incubation in an aqueous solution for 48 h, indicating that CeO_2_ remains firmly immobilized within cSiCe without detachment. According to the standard calibration of ICG *vs*. absorption intensity at 780 nm, the loading efficiency of ICG was calculated as 20.61% (Fig. S4). Meanwhile, we also investigated the stability of ICG within the chiral mesopores of cSiCe&ICG after incubation with serum for different periods. After 72 h of incubation, ICG retained more than 90% of its initial fluorescence intensity (Fig. S5), demonstrating its excellent stability within cSiCe without detectable leakage. Ultimately, cSiCe&ICG was evaluated in buffer solutions that mimic the tumor microenvironment with a pH of 6.5 and physiological conditions with a pH of 7.4. TEM images manifested that biodegradation is substantially more rapid under weak acidic conditions than at neutral pH that the spheres maintained their shape for up to 36 h (Fig. 2K). Under pH 6.5, the structure began to break down after only 6 h, and by 24 h, the particles had fragmented into many small nano-pieces (Fig. 2K). By 36 h, they had completely disintegrated with minimal residual debris, highlighting the highly sensitive response of this chiral mesoporous nanomedicine to acidic conditions. Especially, we measured the release efficiencies of Ce ions and ICG from cSiCe&ICG under the weakly acidic conditions of the tumor microenvironment. The results showed that both exhibited identical time-dependent release profiles (Fig. S6), further confirming the tumor microenvironment-specific responsiveness of cSiCe&ICG. To further study the biodegradation feature, we constructed symmetric Ce-doped dendritic mesoporous silica (mSiCe) and loaded ICG within its mesochannels (mSiCe&ICG) (Fig. S7). Inductively coupled plasma optical emission spectrometer (ICP-OES) analysis of mSiCe revealed that the mass ratio of Ce to Si was also 2:7, which was nearly identical to that in the as-prepared cSiCe. Meanwhile, we measured the X-ray Diffraction (XRD) patterns of the chiral and nonchiral nanoprobes. The two patterns were highly similar and exhibited only a single broad peak (Fig. S8), indicating that both probes possessed amorphous structures. The size and surface negative charge of mSiCe&ICG and cSiCe&ICG were almost identical (Figs. S9 and S10), meanwhile, they also exhibited excellent stability (S11). We found that the chiral structure exhibited a higher degradation rate than the symmetric structure (mSiCe&ICG) (Fig. S12), suggesting that chirality can enhance the reaction efficiency under mildly acidic conditions. These results collectively confirmed the successful synthesis of highly uniform, monodisperse mesoporous cSiCe&ICG with chiral and tumor microenvironment-responsive properties.

**Fig. 2:**
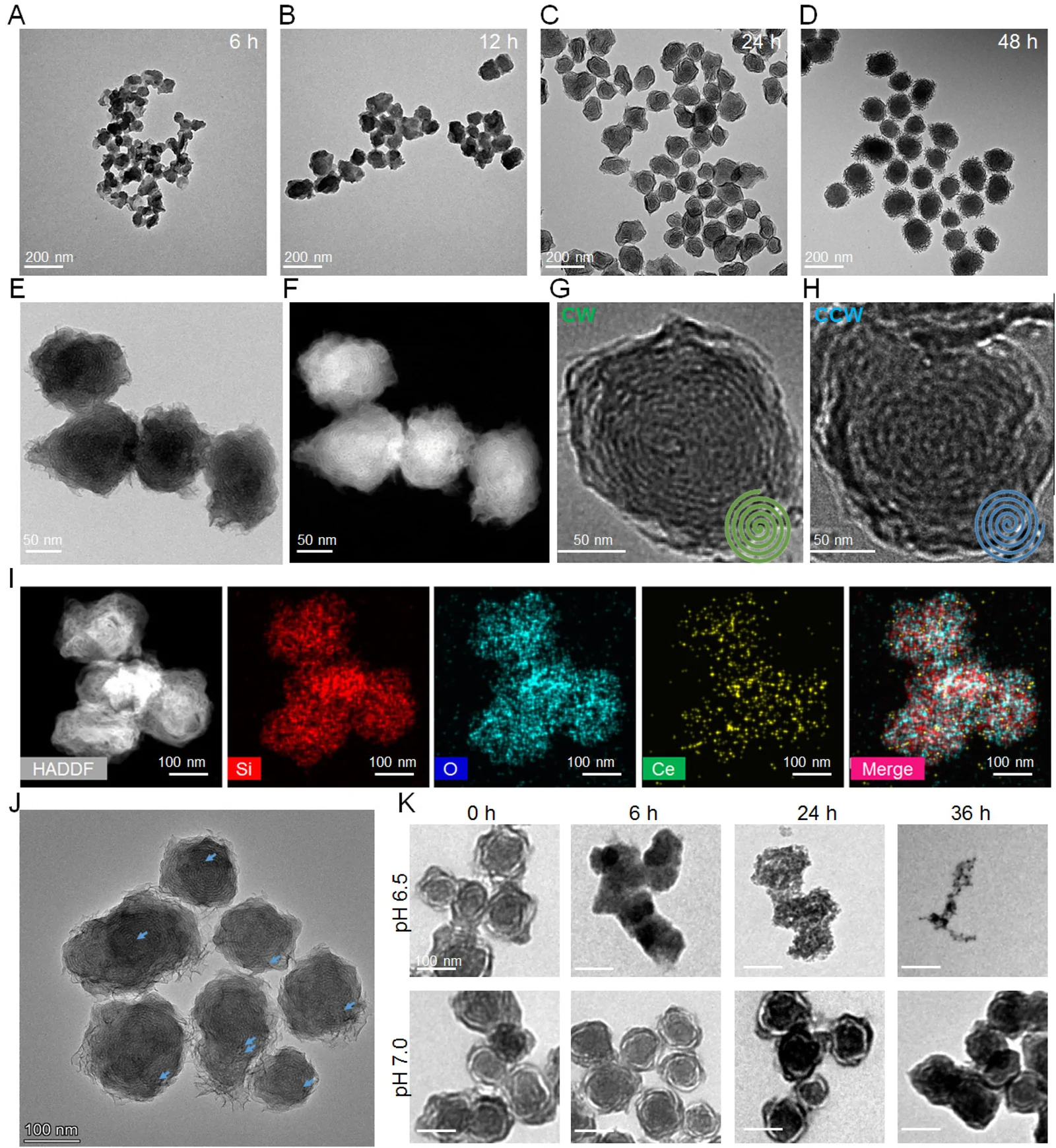
TEM images of cSi nanoparticles after mixing for (A) 6 h, and the progression of cSiCe nanoparticles after mixing for (B) 12 h, (C) 24 h, and (D) 36 h. (E) HRTEM and HAADF-STEM images of cSiCe nanoparticles after 24 h of stirring. (F) HRTEM images and schematic representations of the clockwise (CW) configuration of cSiCe. (G) HRTEM images and schematic representations of the counterclockwise (CCW) configuration of cSiCe. (H) Elemental distribution maps highlighting the unique chiral architecture of cSiCe nanoparticles. (I) HRTEM images of cSiCe&ICG nanoparticles. (J) Time-lapse TEM imaging of cSiCe&ICG nanoparticles suspended in buffers at pH levels of 7.4 and 6.5.

### 3.2 Assessment of photostability, ROS generation, and photothermal effects

The off-peak emission region of ICG within the NIR II spectrum is beneficial for the early and accurate detection of cancerous tissues. Similarly, the emission-spectra of ICG-loaded nonreactor systems successfully extended into the NIR II, with notably strong off-peak NIR II light images for both ICG based samples, confirming the successful synthesis of cSiCe&ICG (Fig. 3A). However, ICG faces challenges such as instability in aqueous environments, susceptibility to photodegradation, and low fluorescence quantum yield under continuous 808 nm laser irradiation, which restrict its use for *in vivo* NIR fluorescence bioimaging. Subsequently, aqueous dispersions of ICG, mSiCe&ICG, and cSiCe&ICG were imaged using an InGaAs camera with a 1000 nm long-pass-filter under continuous natural light exposure. As anticipated, the ICG group displayed rapid fluorescence decay, with no signal detected after 24 h (Fig. 3B and C). Interestingly, we observed a slight increase in fluorescence over time. Encouragingly, the NIR II signal from the cSiCe&ICG group intensified rapidly with time and reaches a plateau at 6 h. Quantitative analysis of fluorescence intensity at each time point reflected that the cSiCe&ICG group was significantly stronger than the mSiCe&ICG group. Specifically, the intensities of these two silica-based groups are 21-fold and 14-fold stronger than that of the ICG sample, respectively (Fig. 3C). Given the novel discovery that chiral structural nanocarriers could significantly enhance ICG fluorescence, we also examined fluorescence variations under different concentration conditions. Within the radially divergent, symmetric mesopores, although fluorescence quenching by water molecules is reduced, ICG tends to form J-aggregates. [47], [48]. The unique chiral helical channels of cSi enhanced the monomeric absorption of ICG [49], in particular, under 808 nm laser irradiation, the increase in temperature further promotes the dispersion of monomeric ICG. A similar phenomenon has also been observed when ICG is associated with proteins in bovine (BSA) and human (HSA) serum albumins [50]. Compared to dendritic mesopores, the chiral mesopores with fewer open ends and multi-shells in a spiral direction further accelerated ICG molecule motion, hence, cSiCe&ICG exhibited more pronounced fluorescence enhancement. Unsurprisingly, the fluorescence enhancement was concentration dependent, indicating that chiral mesoporous silica nanocarriers are advantageous for prolonged intraoperative luminescence imaging (Fig. 3D). Latterly, the valence states of Ce in cSiCe&ICG were characterized by X-ray photoelectron spectroscopy (XPS). The survey spectrum confirmed the presence of Si 2p, O 1s, and Ce 3d signals. The high-resolution Ce 3d spectrum revealed the coexistence of Ce^3+^ and Ce^4+^, with a ratio of approximately 4:3 based on the fitted peak areas (Fig. S13). The relatively high Ce^3+^ content is favorable for the subsequent Fenton-like reaction. We then investigated the chiral catalysis effect of cSiCe&ICG. Employing 3,3,5,5-tetramethylbenzidine (TMB), we quantified the *in vitro* ROS mediation from the cSiCe&ICG nanomedicine. The oxidative product of TMB (oxTMB) exhibited a prominent absorption peak at 645 nm. The cSiCe&ICG formulations were pre-conditioned at a pH of 6.5 for 36 h and subsequently incubated with H_2_O_2_ and TMB. Significantly, the combination of cSiCe&ICG with 808 nm irradiation led to the most pronounced ROS generation compared to mSiCe&ICG + L, as shown in Fig. 3E. Meanwhile, the generation of ROS from chiral CeO_2_ catalysis was concentration-dependent (Fig. 3F). The •OH, which was further determined by an electron spin resonance (ESR)-spin trapping technique, was generated by the addition of cSiCe&ICG or mSiCe&ICG. The •OH generation efficiency of the chiral nanoreactor was significantly higher than that of the achiral nanoreactor (Fig. S14), which was mainly attributed to the ability of chirality to enhance biocatalytic activity [51]. In brief, these results confirmed that the tumor microenvironment stimuli cSiCe&ICG could efficiently produce a hyperthermia-enhanced ROS storm more effectively than traditional symmetric catalysts. Because Ce^4+^ is capable of depleting GSH, additionally, under pH6.5 treatment of 36 h, we also found that cSiCe also presented over 85% GSH depletion effect *via* the indicator 5,5′-dithiobis-(2-nitrobenzoic acid) (DTNB) (Fig. S15). GSH depletion reduces ROS scavenging, thereby facilitating the generation of an ROS storm. This result prompted us to delve into the photothermal capabilities of cSiCe&ICG. As depicted in Fig. 3G and H, a 200 μg/mL solution of cSiCe&ICG under 808 nm laser irradiation at a power density of 0.8 W/cm^2^ reached a temperature of 60.3°C in just 5 min, whereas the same conditions only heated free ICG and PBS to 47.9°C and 30.5°C, respectively. This enhanced performance was primarily due to the chiral mesoporous structure that protected ICG from photodegradation in water, thus improving its photothermal efficiency. Moreover, the 200 μg/mL cSiCe&ICG solution demonstrated excellent photostability over multiple cycles of laser activation and deactivation, as shown in Fig. 3I. Moreover, it also showed a dose-dependent increase in temperature (Fig. 3J). Furthermore, to determine the efficiency of converting light energy into heat (denoted as η), we examined the temperature changes during the laser's activation and deactivation (heating and cooling phases; Fig. 3K). Employing a standard protocol, we found that the light-to-heat conversion efficiency was approximately 58.8% (Fig. 3L). Collectively, these findings suggested that the chiral cerium-doped nanomaterial was a viable contender for PTT, capable of enhancing ROS storm to effectively combat malignant bladder tumors.

**Fig. 3:**
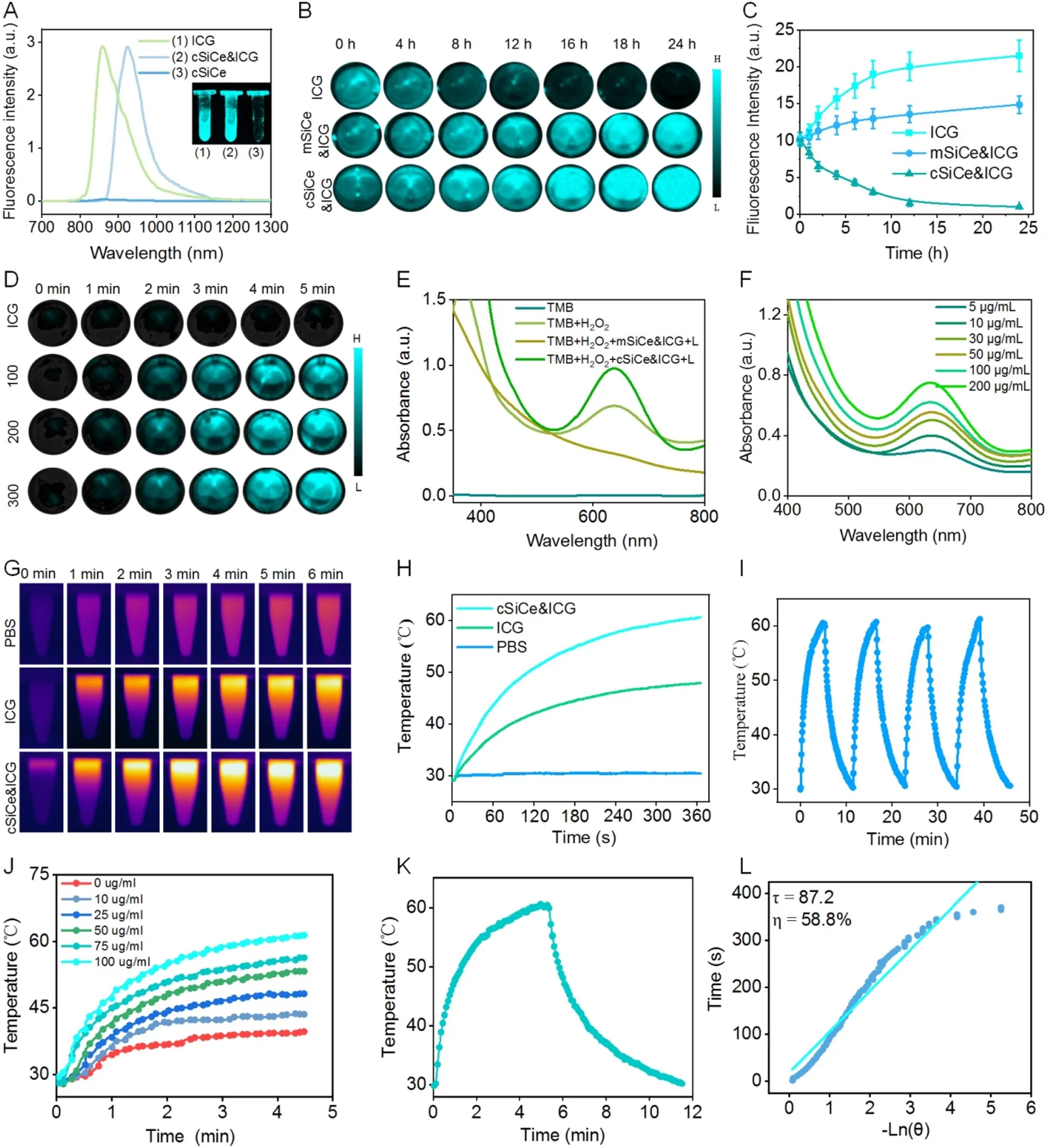
(A) Emission spectra of cSiCe, ICG, and their composite cSiCe&ICG, with NIR-II images shown as inserts. (B) Photographs of NIR II fluorescence, (C) the associated quantitative fluorescence intensities of ICG, mSiCe&ICG, and cSiCe&ICG following prolonged exposure to ambient lighting for different time periods. (D) NIR II fluorescence imaging and (E) the corresponding signal strength of ICG and cSiCe&ICG at various concentrations post-irradiation with an 808 nm laser for a set duration. (F) Absorbance spectra of the oxidized TMB assay under diverse treatment conditions. (G) Absorbance spectra of the oxidized TMB after interaction with varying concentrations of cSiCe&ICG. (H, I) Thermal imaging and temperature profile graphs of cSiCe&ICG, PBS, as well as ICG after illuminated by 808 nm laser irradiation for a 5-min period. (J) Photothermal cycle stability of cSiCe&ICG assessed over multiple laser activation/deactivation sequences. (K) Temperature elevation profiles of cSiCe&ICG with various doses upon 808 nm laser exposure for 5 min. (L) The temperature change of cSiCe&ICG during continuous laser irradiation and its subsequent cooling after the laser is turned off. (M) Linear regression analysis of the natural logarithm of θ in relation to cooling time.

### 3.3 Cellular uptake and enhanced anticancer effects *in vitro*

For nanomedicines to effectively combat tumors, their internalization by cancer cells is essential. To study the cellular uptake of cSiCe&ICG, we utilized CLSM under 633 nm laser excitation to observe the process. In this experiment, the bladder cancer cell line MB49 was incubated with cSiCe&ICG for various durations. The CLSM photographs revealed that the red light from ICG within the tumor cells increased with time, peaking after 8 h of incubation (Fig. 4A). Interestingly, according to CLSM and flow cytometer results, cSiCe&ICG displayed a significantly higher rate of cellular uptake compared to mSiCe&ICG and ICG alone (Fig. 4C–S16, S17). Encouragingly, we also observed enhanced cellular uptake of cSiCe&ICG compared with conventional mSiCe&ICG in other cancer cell lines, including colon and breast cancer cells (Fig. S18), suggesting that the enhanced cellular uptake of nanomaterials with chiral structures may be broadly applicable across different cancer cell types. To further evaluate the cellular uptake of the chiral particles, normal cells were incubated with cSiCe&ICG or mSiCe&ICG, and the intracellular Ce content was quantified by ICP-OES. The results demonstrated that the chiral particles also displayed high uptake efficiency in normal cells (human umbilical vein endothelial cells, HUVECs) (Fig. S19). This enhanced uptake was consistent with previous report [52], which suggests that the unique chiral structure of cSiCe&ICG facilitated more efficient cellular internalization in cancer cells. Using specific endocytosis inhibitors, we found that the cellular uptake of both chiral and nonchiral nanoprobes was energy-dependent (Fig. S20A and B). However, the chiral probes were internalized primarily through both caveolin- and clathrin-mediated pathways, whereas the nonchiral nanoprobes entered cells mainly *via* the caveolin-mediated pathway (Fig. S20C and D). Consequently, the cellular uptake of cSiCe&ICG was significantly enhanced. ROS, including superoxide, hydroxyl radicals, as well as singlet oxygen, can be quantified using DCFH-DA, a common *in vitro* ROS indicator. The intensity of green fluorescence (excitation at 488 nm and emission at 550 nm) indicated the amount of ROS present in the cytoplasm of the tumor cells. MB49 cells treated with cSiCe&ICG alone exhibited a faint green signal, indicating low ROS production. However, upon exposure to 808 nm light, these cells show a significantly stronger fluorescence compared to the mSiCe&ICG + L group (Fig. 4B–S21). Besides, as shown in Fig. S22, flow cytometric analysis of DCFH-DA fluorescence intensity in each group manifested a trend consistent with the CLSM results. Given the CDT effect of cSiCe&ICG, we utilized hydroxyphenyl fluorescein (HPF), a commercial indicator for •OH, to observe the production of •OH within the cytoplasm. The group treated with cSiCe&ICG and exposed to 808 nm light exhibited a significantly enhanced green fluorescence, confirming the superior ROS-based CDT potential of the chiral mesoporous nanocatalyst over the dendritic counterpart when activated by NIR laser (Fig. 4E–S23). Increased ROS could overwhelm cellular antioxidants, leading to mitochondrial DNA damage and dysfunction, thus enhancing oxidative stress. To investigate mitochondrial oxidation mediated by cSiCe&ICG, the MMP was assessed by the JC-1 probe. It produced red signal aggregates in healthy mitochondria and green signal monomers in damaged mitochondria. A transition from red to green fluorescence suggested a reduction in mitochondrial membrane potential and extensive mitochondrial injury. After incubation for 8 h, the PBS group manifested minimal green signal, indicating healthy mitochondria, while cSiCe&ICG treatment could lead to a red fluorescence decrease and a green fluorescence increase, indicating mitochondrial damage. Moreover, the cSiCe&ICG + L group displayed a more pronounced green light than the mSiCe&ICG + L group with significantly lower red to green fluorescence ratio, suggesting greater mitochondrial dysfunction induced by the chiral nanocatalyst (Fig. 4D–S24). The JC-1 staining data *via* flow cytometer further confirmed that local hyperthermia pronouncedly amplified the oxidative efficiency from ROS (Fig. S25). To verify the observations, we evaluated the cell killing ability of cSiCe&ICG on HUVECs. Even at the highest concentration (300 μg mL^−1^) of cSiCe&ICG, 93.8% of health cells remained alive (Fig. 4F). Because cSiCe&ICG is responsive to the tumor microenvironment, negligible Ce ion release occurs in normal cells, resulting in low cytotoxicity. Importantly, for bladder tumor cell killing, the collaboration of cSiCe&ICG and an 808 nm light mediated a predominant decrease in cell viability, with nearly a 2.4-fold (29.1%) reduction in live cells compared to cSiCe&ICG alone (69.6%). Encouraged by the significant cytotoxicity observed in the cell viability assays, we proceeded to quantify the rate of apoptosis in MB49 cells. Post-exposure to different treatments, the tumor cells were subjected to dual staining with calcein AM and propidium iodide (PI). Consistent with expectations, the apoptotic and necrotic rates were minimal (2.8%) in MB49 cells that were treated with PBS. The sole CDT group (cSiCe&ICG) had a similar cytotoxic effect. Notably, the apoptosis/necrosis investigation presented that cSiCe&ICG + L administration induced the most favorable therapeutic outcome, with approximately 62.5% apoptotic cell death (Fig. 4G–J). Furthermore, we employed CLSM to detect the green and red emissions that correspond to living and dead cells, respectively. As evidenced in Fig. 4I and K, there was no significant damage observed in the PBS group. In contrast, 47.6% of MB49 cells were found to be alive in the mSiCe&ICG + L group, whereas only 31.2% of the cells survives after exposure to cSiCe&ICG + L. These results underscored the precision of cSiCe&ICG nanomedicine in targeting tumors and its enhanced potency in fighting cancer when activated by laser irradiation, surpassing the performance of mSiCe&ICG. Consequently, cSiCe&ICG emerged as a potential candidate for a synergistic photothermal therapy/reactive oxygen species (PTT/ROS) nanomedicine, showing promise for the clinical management of bladder tumors.

**Fig. 4:**
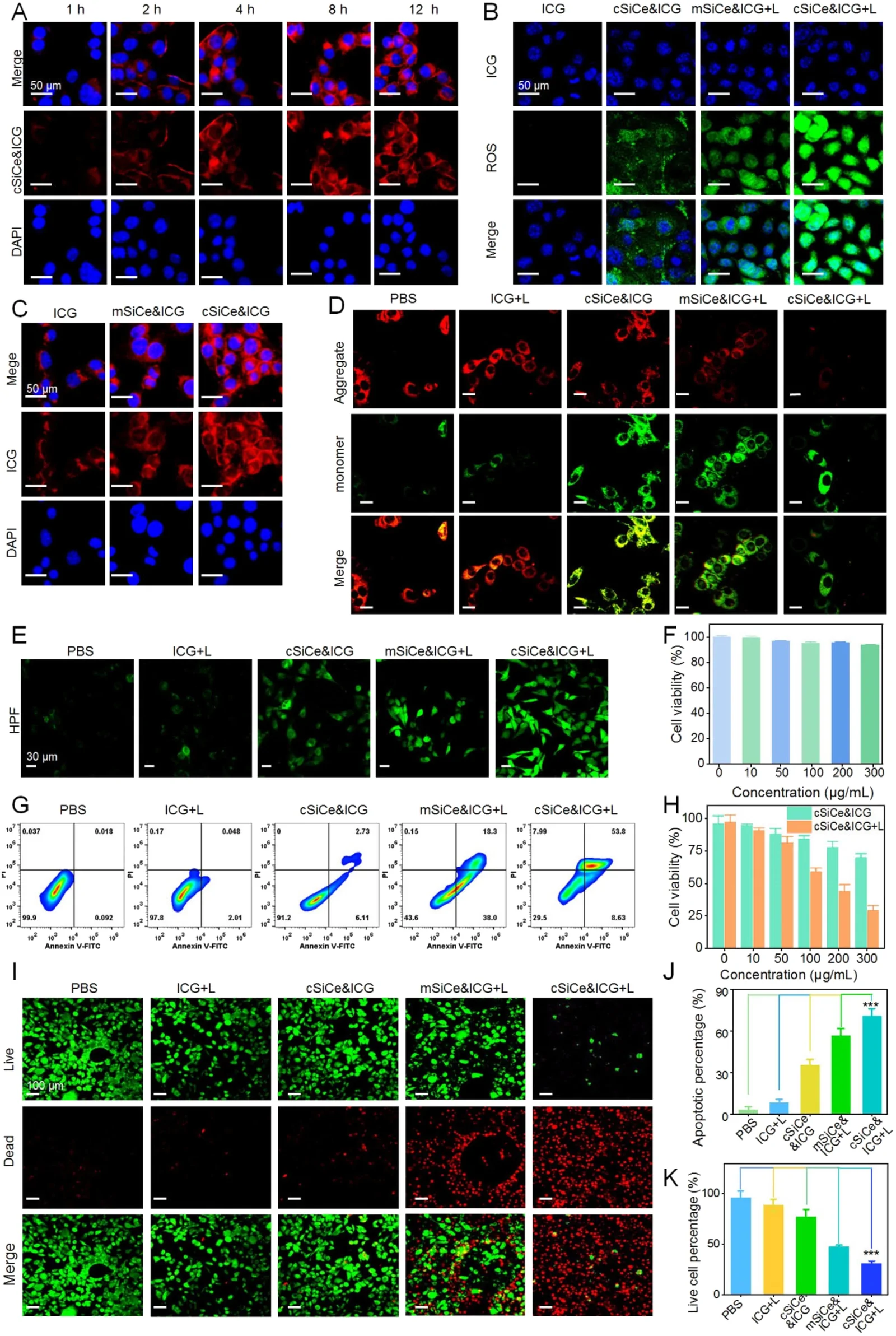
(A) CLSM visualization of MB49 cells interacting with cSiCe&ICG over various time intervals. (B) Detection of intracellular ROS in MB49 cells following distinct treatments-namely ICG, cSiCe&ICG, mSiCe&ICG + L, and cSiCe&ICG + L after induction with H_2_O_2_. (C) CLSM captures of MB49 cells post-incubation with ICG, mSiCe&ICG, and cSiCe&ICG. (D) CLSM visualization of MB49 cells analysis by JC-1 following exposure to PBS, ICG + L, cSiCe&ICG, mSiCe&ICG + L, and cSiCe&ICG + L. (E) HPF stained tumor cells after various treatments. (F) Assessment of the cytotoxic effects of cSiCe&ICG toward normal cells (HUVECs) at diverse doses for a 24 h. (H) Analysis of the comparative efficacy of cSiCe&ICG alone or combined with laser stimulation in the elimination of MB49 cells. (G) Examination of cell-death pathways *via* flow-cytometry and (J) determination of apoptosis ratios of MB49 cells after administrated with PBS, ICG + L, cSiCe&ICG, and cSiCe&ICG + L. (I) Measurement of tumor cell viability using fluorescence-based methods and (K) assessment of live cell counts post-treatment. Significance levels are indicated by ** (p < 0.01), with error bars representing standard deviation (n = 4).

### 3.4 Targeted BC localization and *in vivo* photothermal effects

Encouraged by the robust *in vitro* carcinoma tissues targeting and therapeutic capabilities of cSiCe&ICG, we proceed to evaluate its tumor enrichment and the subsequent PTT effect *in vivo*. We first assessed the bio-distribution and BC targeting in BALB/c mice with MB49 bladder tumors under the observation of NIR II fluorescence imaging. Following intravenous administration of ICG, mSiCe&ICG, or cSiCe&ICG, the animal were subjected to imaging under a small fluorescent imaging system with 808 nm laser shining and with a 1000 nm long-pass-filter. Both two Ce based formulations gradually accumulated in the tumor tissues, with cSiCe&ICG showing a higher NIR II light intensity than mSiCe&ICG acrossed all post-injection time period, confirming a preference for internalization *via* bladder tumor cells, consistent with *in vitro* endocytosis of MB49 cells (Fig. 5A). This augmented uptake might be due to the asymmetric and chiral nanostructure, which promoted specific tumor cellular internalization (Fig. 5A). The tumor outline was clearly visible from 6 to 12 h post-injection with cSiCe&ICG, with the fluorescence signal diminishing to faint by 24 h post-injection. On the other hand, free ICG exhibited a negligible fluorescence at the tumor site throughout the imaging period. Significantly, ICP-OES was applied for measuring the Ce ions in tumor sites from the two nanoformulations. As shown in Fig. 5B, the Ce ion concentration at the tumor site for the chiral probe group was overwhelmingly higher than that of the achiral (symmetric) probe group before 48 h post-injection, highlighting the superior tumor-targeting ability of the chiral probes. The TBR values from the cSiCe&ICG treat mice were considerably higher than the values from the mSiCe&ICG or ICG groups (Fig. 5C). In the cSiCe&ICG group, the TBR remained nearly unchanged from 6 to 12 h, indicating the excellent tumor retention of the chiral probe. To investigate the underlying mechanism, bladder tumor-bearing mice were intravenously injected with mSiCe&ICG or cSiCe&ICG. At 12 h post-injection, tissue sections were collected from the central region of the tumors, and the ICG fluorescence signals were examined by fluorescence microscopy. Strikingly, the fluorescence intensity in the cSiCe&ICG group was markedly higher than that in the mSiCe&ICG group (Fig. S26), demonstrating the superior tumor tissue penetration of the chiral nanomaterial. This enhanced penetration likely reduces intratumoral clearance, thereby contributing to improved retention at the tumor site. The cooperate effect of the increased cellular endocytosis of tumor cells as well as the enhanced permeability and retention (EPR) effect of BC tissues might be due to the unmatched chiral mesoporous nanostructure of cSiCe&ICG markedly improved tumor cell recognition and enrichment, with maximal accumulation occurring 12 h after administration with highest TBR a in the cSiCe&ICG group, approximately 10.8 times higher than in the ICG treated mice. Accordingly, this time point was chosen for the early diagnosis of bladder tumors and for photoactivated therapy. Accordingly, local tumor temperatures in the three groups were measured accompanying by 808 nm laser irradiation after injection for 12 h. In the cSiCe&ICG group, the temperature rapidly increase to nearly 60.2°C after continuous irradiation *via* a laser for 5 min, higher than that of mSiCe&ICG (52.9°C). Under identical conditions, the temperature in the PBS-treated group rose only to about 33.7°C (Fig. 5D and E). Overall, our chiral nanoreactor cSiCe&ICG, featuring precise tumor targeting, provides improved diagnostic performance and potent combined therapeutic effects.

**Fig. 5:**
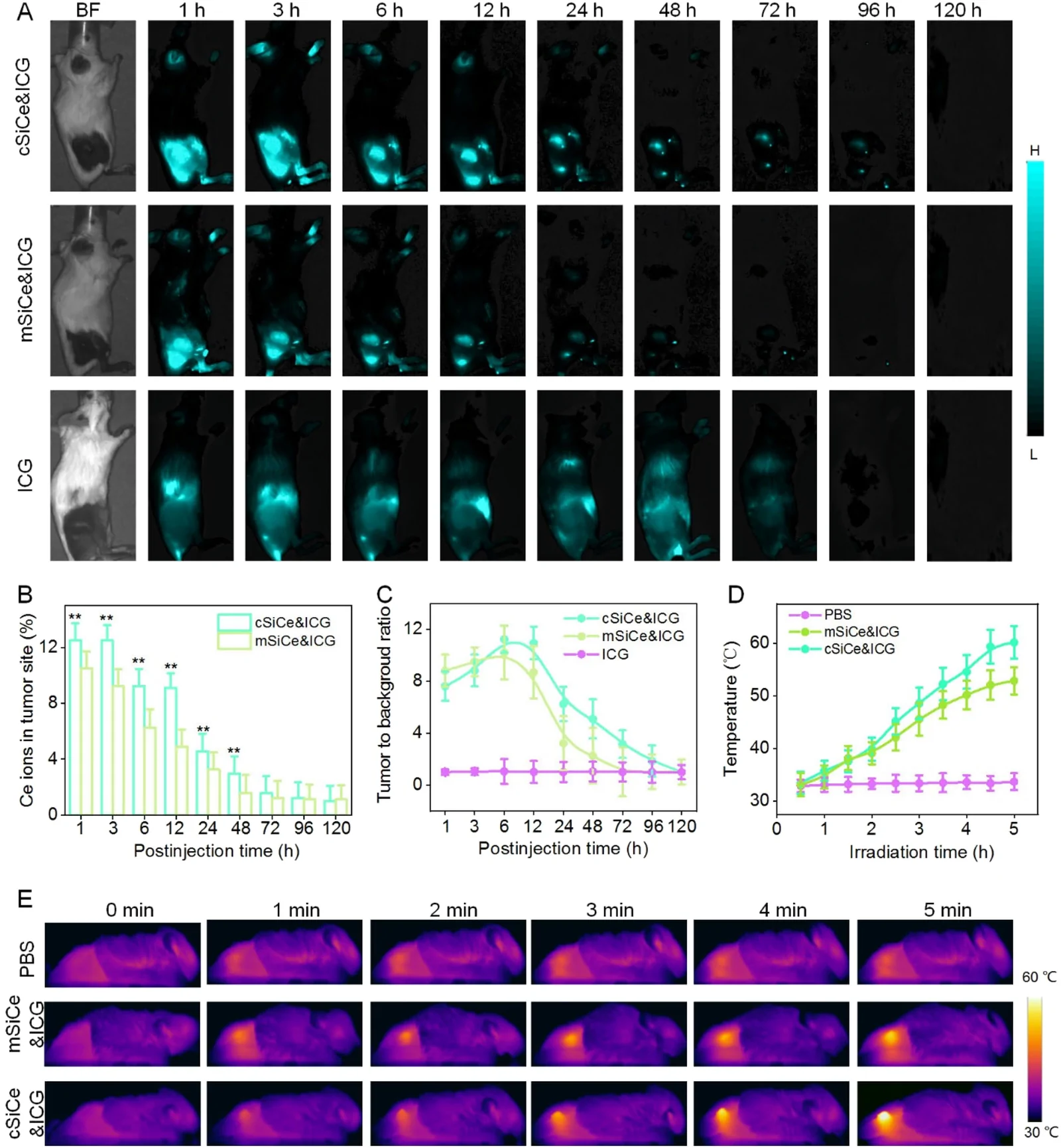
(A) NIR II fluorescent imaging of mice with subcutaneously implanted bladder tumors at different time intervals post intravenous injection of cSiCe&ICG, mSiCe&ICG, and ICG through the tail vein. (B) Concentration of Ce ions in tumor tissues over various h following administrations of cSiCe&ICG, mSiCe&ICG, and ICG. (C) TBR in mice after intravenous injection of cSiCe&ICG, mSiCe&ICG, or ICG for various h. (E) Visual documentation of photothermal effects and (D) corresponding graphs illustrating temperature elevations in the tumor region during continuous 808-nm laser irradiation over different durations following the 12 h post-injection administration of cSiCe&ICG, mSiCe&ICG, and ICG. ** denotes statistical significance at P < 0.01. The data are expressed as mean ± standard deviation (n = 4).

### 3.5 Blood circulation and fluorescence-navigated tumor surgery

Chiral nanoparticles have demonstrated great prospect for efficient protein corona assembly and extended circulation times in the bloodstream. Sodium Dodecyl Sulfate-Polyacrylamide Gel Electrophoresis (SDS-PAGE) was employed to verify the protein corona formation around conventional mSiCe&ICG and chiral cSiCe&ICG nanoparticles. After staining with Coomassie Brilliant Blue, visible protein bands indicated serum protein adsorption. Of note, within the 3 to 9-h incubation period, the cSiCe&ICG nanoparticles presented a significantly richer protein corona compared to the dendritic mesoporous mSiCe&ICG, as indicated by more intense bands (Fig. 6A–C). Encouraged by these results, we examined the presence of Ce ions in blood samples from these two groups. Our findings revealed that cSiCe&ICG had a longer half-life in the bloodstream at 11.7 h compared to mSiCe&ICG at 7.4 h (Fig. 6D). The enhanced blood retention and protein corona formation of cSiCe&ICG enabled it to avoid rapid clearance by the reticuloendothelial system (RES), which was advantageous for intraoperative imaging in clinical settings. Based on these outcomes, we studied the bio-distribution within the RES. *Ex vivo* NIR-II light imaging of major organs and tumors following administration of cSiCe&ICG, mSiCe&ICG, and ICG at 12 h post-injection confirmed that the cSiCe&ICG group exhibited the highest tumor accumulation with minimal RES organ uptake compared to other groups (Fig. 6B). The mean fluorescent intensities of *ex vivo* NIR II signals obtained from tissues of organs and tumors further demonstrated that, at this time point, the tumor in the cSiCe&ICG group exhibited significantly higher signal intensity compared to both other organs and tumor groups in the remaining experimental groups (Fig. S27). Based on the TBR and peak accumulation data, this time point was determined to represent the optimal window for NIR-II fluorescence imaging-guided bladder tumor resection. Excitingly, 12 h post-injection of cSiCe&ICG, NIR II fluorescent imaging clearly delineated the tumor margins (Fig. 6F). The primary tumor mass (T1) was first excised under white light illumination, after which the NIR II imaging system was employed to scrutinize the tumor margins for any residual fluorescence signals. Guided by these fluorescence signals, any remaining tissue (T2) identified was subsequently resected with precision. Moreover, muscle tissue in the surgical bed was resected after tumor removal to assess for any postoperative remnants. H&E staining of T1 and T2 revealed well-defined boundaries between tumor and normal tissue, with no residual BC cells detected in the surrounding surgical bed (Fig. 6H). By contrast, after solid tumor was resected under white light, normal tissue (white light-surgical bed, Fig. 6I) from the resected tumor tissues revealed residual tumor cells upon H&E staining, highlighting the limitations of white light-guided tumor resection in clinic surgery. At 30 days post-surgery, tumor recurrence and metastasis were evaluated by bioluminescence imaging following intravenous administration of D-luciferase potassium, which elicited green luminescence from any remaining MB49-luc cells. Particularly, no bioluminescent signal was observed in the NIR II fluorescence imaging-guided surgery group (Fig. 6G), the quantitative results showed that the photon flux in the white-light group was significantly higher than that in the cSiCe&ICG group (Fig. S28), demonstrating the absence of tumor recurrence and all mice in the white light group represented signs of recurrence. At the same time, we also found that after tumor resection in the NIR II light bioimaging group, the survival rate reached 80% within 60 days, which was prominently higher than the white light group (10%), demonstrating the high accuracy of the cSiCe&ICG probe in tumor fluorescence imaging (Fig. 6E). Collectively, these results verified that the tumor microenvironment-responsive cSiCe&ICG nanoprobes were capable of accurately guiding total tumor resection, thereby ensuring the precise and thorough elimination of all carcinoma tissue *in vivo*.

**Fig. 6:**
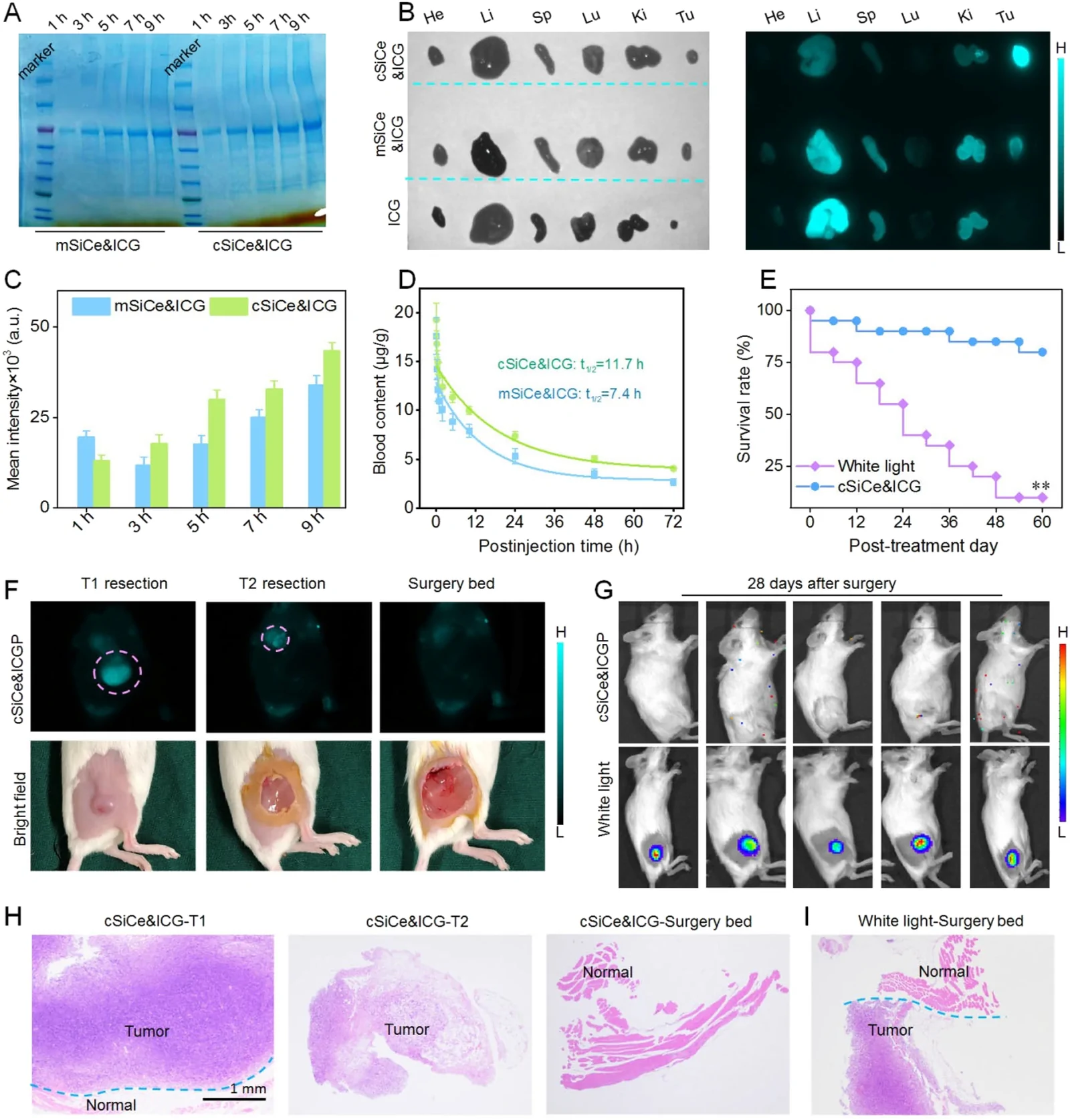
(A) Gel electrophoresis analysis illustrating protein-corona formation and (C) quantitative evaluation the intensities of corresponded band upon incubation of BSA with cSiCe&ICG and mSiCe&ICG at varying time intervals. (B) *Ex vivo* NIR II fluorescent images of major organs and tumor tissues harvested from cSiCe&ICG and mSiCe&ICG treated mice. (D) Circulation times of cSiCe&ICG and mSiCe&ICG in blood at various intervals. (E) Survival rate curves monitoring for 60 days after tumor surgery under white light and NIR-II fluorescence imaging. (F) Photographs and corresponded NIR II light images depicting a BC-bearing mouse at pre-surgery, first tumor resection (T1), and second tumor resection (T2). (G) Bioluminescence imaging was employed to assess tumor recurrence in bladder tumor-bearing mice at 30 days following surgery performed under either NIR-II fluorescence imaging guidance with cSiCe&ICG or conventional white light illumination. (H) H&E staining evaluation of tissues from T1, T2, and the normal tissues from surgical bed of cSiCe&ICG group. (I) H&E-stained normal tissue from the surgical bed of white light group. The data are presented as mean ± standard deviation (n = 4).

### 3.6 Synergistic *in vivo* suppression of BC cells

After surgery, patient with early-stage BC is generally stratified into high-risk or intermediate-risk group according to pathological evaluation results. Post-surgical adjunctive treatment with cSiCe&ICG, which combines PTT and ROS generation, is anticipated to be effective in suppressing recurrence of bladder tumors in medical practice. We next sought to investigate the *in vivo* therapeutic potential of cSiCe&ICG. BALB/c mice with induced bladder tumors were randomly divided to four treatment groups: (1) PBS, (2) cSiCe, (3) mSiCe&ICG, (4) cSiCe&ICG. At 12 h post-injection, mice in all groups were exposed to 808-nm light irradiation at a power density of 1.5 W cm^−2^ for duration of 5 min. Tumor volumes and body weights of all mice were carefully monitored throughout the study. The PBS + L group displayed rapid tumor progression, whereas the cSiCe + L group exhibited a modest suppression of tumor growth rate, which could be ascribed to the ROS-induced antitumor effects of the Ce-based catalysts (as seen in Fig. 7A–D, F). Notably, the synergistic therapy combining PTT and CDT resulted in a significantly higher regression of tumors over the 21-day treatment period (as depicted in Fig. 7A–D, F). Specifically, the cSiCe&ICG + L treatment significantly outperformed the mSiCe&ICG + L group in tumor suppression, potentially due to the enhanced tumor targeting capabilities of the chiral nanoreactors. At day 21, tumors were harvested and weighed from all experimental groups (as shown in Fig. 7B and C). The cSiCe&ICG combination treatment demonstrate superior therapeutic outcomes compared to the other groups, particularly in terms of photothermal and potent ROS therapy. Furthermore, histological examinations comprising H&E, TUNEL, and Ki67 staining (as depicted in Fig. 7G), indicated that the PTT and enhanced ROS therapy from cSiCe&ICG led to more extensive tumor destruction compared to mSiCe&ICG and the other two groups with only ROS generation, leading to substantial apoptosis and necrosis. To evaluate the biosafety and clinical translational potential of cSiCe&ICG + L, *in vivo* toxicity was assessed by monitoring body weight variations and conducting histological analyses. Throughout the study, mice treated with the nanomedicine exhibited neither significant body weight fluctuations nor histological abnormalities when compared to the PBS control group (as shown in Fig. 7B–S29). Furthermore, blood chemistry indicators and hematological indexes in the cSiCe&ICG-treated mice remained within healthy ranges at 1 day and 2 weeks post-tumor inhibition (as indicated in Figs. S30 and S31). Furthermore, to evaluate long-term toxicity to the reticuloendothelial system, cSiCe&ICG was intravenously injected into healthy mice *via* the tail vein, and the residual Ce and Si ions in the liver, spleen, and lungs were measured at 1, 2, and 4 weeks. The results showed that, after 28 days, both ions had been almost completely cleared from the reticuloendothelial system (Fig. S32), further demonstrating the excellent biosafety of the chiral nanoprobe. Finally, to determine whether cSiCe&ICG induces cytokine associated with immune activation, the serum levels of IL-10, TNF-α, and IFN-γwere detected. No significant fluctuations were observed (Fig. S33) in cSiCe&ICG treated mice. Taken together, these preliminary toxic assessments confirmed that cSiCe&ICG does not mediate appreciable toxic effects, underscoring its potential as a biocompatible nanomedicine for malignant tumor eradiation. In a word, these results suggest that chiral nanoagents capable of augmenting CDT in combination with PTT represent promising candidates for clinic translation in the management of malignant carcinoma.

**Fig. 7:**
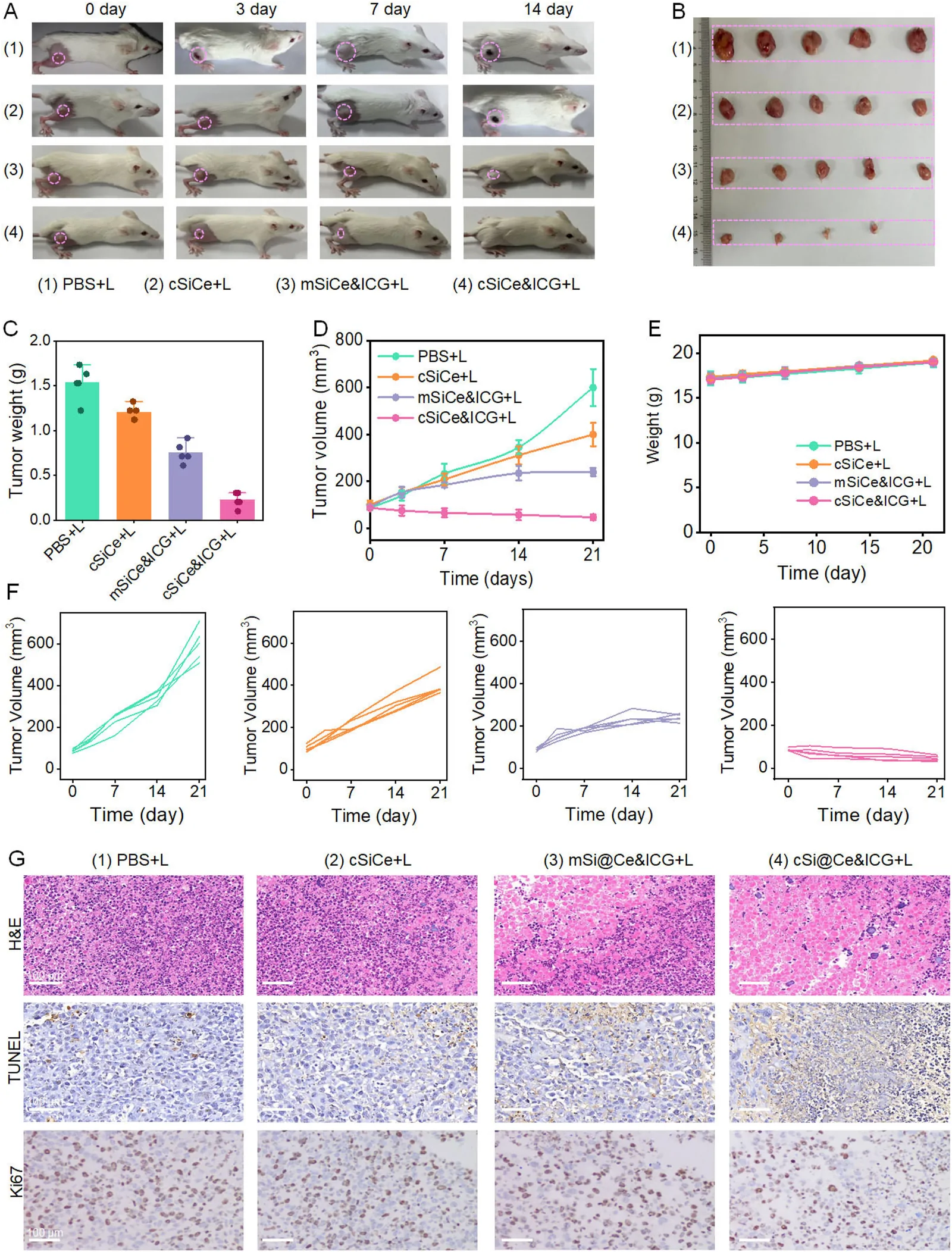
(A) Representative photographs of bladder carcinoma-bearing mice from four distinct treatment groups captured at various time points during the course of tumor therapy. (B) Corresponding isolated tumor samples and (C) the tumor weights following the 21-day treatment phase for each experimental set. (D) Time-dependent tumor volume changes across all treatment groups, along with (F) detailed tumor growth measurements encompassing four groups: PBS + L, cSiCe + L, mSiCe&ICG + L, and cSiCe&ICG + L. (E) Body weight fluctuation of all mice undergoing various therapeutic treatments over time. (G) Histological evaluation of resected tumors *via* H&E, TUNEL, and Ki67 staining. ***P < 0.01 reflects statistically significant differences. Data are presented as mean ± standard deviation (n = 4).

## 4 Conclusion

In this work, we have synthesized uniform chiral cSiCe&ICG nanoparticles with precisely controlled mesoporous structures and a distinct chiral framework, utilizing an established lamellar micelle spiral self-assembly technique. Our findings demonstrated that the cSiCe&ICG nanoparticles possessed exceptional resistance to fluorescence fading, with a notable increase in fluorescence intensity observed even after extended exposure to light. The key features of this nanoplatform include enhanced tumor accumulation, facilitated by the EPR effect and the chiral nanostructure, which promoted endocytosis and improves the nanoreactor's tumor targeting efficacy. Importantly, this tumor acid-responsive nanoagent was instrumental in accurately guiding intraoperative resection of bladder tumors. Furthermore, the nanoreactor provided a programmable therapeutic response that synergistically combines PTT-mediated localized hyperthermia with cerium ion catalysis-induced ROS oxidative stress, thereby targeting the tumor through multiple therapeutic mechanisms simultaneously. The straightforward synthesis of our SiCe&ICG nanoreactor not only aided in guiding surgical tumor removal but also demonstrated a potent synergistic inhibitory effect on solid tumors. This innovative nanotherapeutic held significant promise for rapid clinical translation and enhancing the clinical management of bladder cancer.

## CRediT authorship contribution statement

**Guangwei Zheng:** Validation, Software, Data curation, Conceptualization. **Ao Wang:** Visualization, Software, Formal analysis. **Yiqing Du:** Visualization, Formal analysis. **Caipeng Qin:** Software, Funding acquisition, Data curation. **Yuxuan Song:** Writing – review & editing, Visualization. **Jilin Wu:** Software, Formal analysis. **Xing Luo:** Validation, Software. **Xiaolong Liu:** Writing – review & editing, Visualization, Supervision, Investigation. **Peiyuan Wang:** Writing – original draft, Software, Data curation, Conceptualization. **Tao Xu:** Writing – review & editing, Supervision, Project administration, Formal analysis, Conceptualization.

## Declaration of competing interests

The authors declare that they have no known competing financial interests or personal relationships that could have appeared to influence the work reported in this paper.

## Data Availability

Data will be made available on request.
