# Supplementary material for: Chiral cerium doped nanoreactor-mediated bladder carcinoma reinforced ablation via fluorescence imaging navigated surgery along with photothermal strengthened ROS storm
Source: Mater Today Bio. 2026 Oct 9;41:103746. doi: 10.1016/j.mtbio.2026.103746 (PMC13679373; doi:10.1016/j.mtbio.2026.103746)
Supplement: Supplementary file 1 [file mmc1.docx]

Chiral Cerium Doped Nanoreactor-Mediated Bladder Carcinoma Reinforced Ablation *via* Fluorescence Imaging Navigated Surgery Along with Photothermal Strengthened ROS Storm

Guangwei Zheng^1^, Ao Wang^2^, Yiqing Du^1^, Caipeng Qin^1^, Yuxuan Song^1^, Jilin Wu^1^, Xing Luo^1^, Xiaolong Liu^2,3,*^, Peiyuan Wang^2,*^, Tao Xu^1,*^

1. Department of Urology, Peking University People's Hospital, Beijing 100044, P. R. China.
2. The United Innovation of Mengchao Hepatobiliary Technology Key Laboratory of Fujian Province, Mengchao Hepatobiliary Hospital of Fujian Medical University, Fuzhou 350025, P. R. China.
3. Fujian Institute of Research on the Structure of Matter, Chinese Academy of Sciences, Fuzhou 350002, P. R. China.

Corresponding authors: [xiaoloong.liu@gmail.com](mailto:xiaoloong.liu@gmail.com) to X. Liu, [wangpeiyuan@fjirsm.ac.cn](mailto:wangpeiyuan@fjirsm.ac.cn) to P. Wang, xutao@pkuph.edu.cn to T. Xu

**Part 1: Supplementary Experimental Section**

**1. Materials and characterization**

Hexadecyltrimethylammonium bromide (CTAB), CeCl_3_•6H_2_O, and hexadecyltrimethylammonium p-toluenesulfonate (CTAT) was purchased from Sigma-Aldrich. Cyclohexane, dimethyl sulfoxide (DMSO) and ethanol were obtained from Shanghai Chemical Co., Ltd. Tetraethyl orthosilicate (TEOS) and triethanolamine (TEA) were purchased from Aladdin Industrial Inc. PBS and NaOH and from Aladdin Industrial Inc. All chemicals were used as received without further purification ICG, JC-1, 2',7'-Dichlorodihydrofluorescein diacetate (DCFH-DA), DAPI were purchased from Beyotime Biotechnology. Deionized water was used in all experiments.

Transmission electron microscopy (TEM) was performed using a JEM 2100F instrument (Japan) operating at an acceleration voltage of 200 kV. High-resolution TEM and energy-dispersive X-ray spectroscopy (EDS) mapping were conducted on a Bruker Multimode 8 high-resolution transmission electron microscope, which was operated at 200 kV, along with a LEO1530VP SEM (Germany). Scanning electron microscopy (SEM) analysis was carried out using a Nanoscope V multimode atomic force microscope. Prior to the measurements, the samples were first suspended in ethanol and then transferred onto copper grids coated with a thin carbon film. Fourier transform infrared (FTIR) spectra were recorded using a Thermo Nicolet iS50 spectrometer. X-ray diffraction (XRD) patterns were obtained with a Rigaku Miniflex 600 system. UV-visible absorption spectra were measured using an Agilent Cary 5000 spectrometer, covering a wavelength range from 0 nm to 2000 nm. Unless otherwise stated, all spectral data were collected under consistent experimental conditions. Fluorescence imaging of tumor cells was performed using a Nikon A1RMP confocal laser scanning microscope. Infrared thermographic images were captured using an FOTRIC 225s infrared camera. In vivo second near-infrared (NIR II) fluorescent imaging was conducted using a NIR-OPTICS Series III 900/1700 system, with excitation provided by an 808 nm laser and a 1000 nm long-pass filter.

**2. Fabrication of mSiCe&ICG**

Mesoporous silica nanoparticles with CeO_2_ doping were synthesized using TEOS as a silica source and CTAB as a surfactant. First, 0.75 g CTAB and 45 mg of CeCl_3_•6H_2_O were added into a round-bottom flask containing 60 mL of ultrapure water, dissolved by ultrasound, heated in an oil bath pot at 60 °C, and stirred. Thereafter, 0.75 mL of 0.1 mol/L NaOH solution was added, followed by 20 mL of a TEOS and cyclohexane mixture. After bathing for 72 h, the mixture was centrifuged at 10000 rpm for 10 min, and the precipitate was collected and washed for several times.

The final mSiCe&ICG composite was fabricated through the following procedure: Initially, 1 mg of ICG powder was dissolved in 10 mL of distilled water. Then, 10 mL of a 1 mg/mL mSiCe aqueous solution was mixed with the ICG solution. The mixture was vigorously stirred at room temperature overnight to allow efficient adsorption of ICG molecules into the mesoporous structure of mSiCe. Following centrifugation, the sample was washed three times with water to remove any unbound ICG. Finally, the mSiCe&ICG nanoplatform was re-suspended in 5 mL of water for further use.

**3. Photothermal conversion efficiency estimation**

The η value (photothermal conversion efficiency) of cSiCe&ICG was determined as follows: 200 μg/mL of mSiCe&ICG were added into the DI water and irradiated by 808 nm laser (0.8 W/cm^2^, 5 min). The temperature curve during laser on/off was measured and η can be apprised by the following two equation.

$\eta=\frac{hA\triangle T_{max}-Q_{s}}{I (1-{10}^{{-A}_{\lambda}})}$	(1)

$\tau=\frac{m_{D}c_{D}}{\mathrm{hA}}$	(2)

In equation (1), _△_Tmax represents the maximum temperature achieved under steady-state conditions, which is 60.2°C for cSiCe&ICG (as shown in Figure 3H). Qs refers to the heat loss due to water evaporation, and its value is calculated as 0.00822. I used a laser power density of 0.8 W/cm² at 808 nm, and Aλ denotes the absorbance of cSiCe&ICG at this wavelength, which was measured as 0.325. From these values, the only unknown parameter hA can be determined using equation (1).

In equation (2), mD is the mass of water, which is 0.2 g, cD represents the specific heat capacity of water, with a value of 4.2 J/gK, and τ is the time constant associated with heat transfer, which was found to be 87.2 seconds (as indicated in Figure 3L). Using this equation, the value of hA is calculated as 1.8×10^-3^ W/K. Based on these results, the efficiency η can be determined to be 58.8%.

**4. Cytotoxicity estimation of cSiCe&ICG**

All experiments were conducted in a 96-well plate. The cytotoxicity of cSiCe&ICG was evaluated using the MTT assay. Briefly, MB49 cells were obtained through enzymatic digestion and seeded into the wells at a density of 5×10^3^ cells per well in 100 μL of DMEM containing 10% fetal bovine serum (FBS), 100 units/mL penicillin, and 100 μg/mL streptomycin. The cells were then incubated at 37°C for 24 hours. Afterward, MPLa&ICG at different concentrations was introduced, followed by an additional 8-hour incubation period. Subsequently, 10 μL of MTT solution dissolved in 1 mL fresh culture medium was added to each well. Following a 2-hour incubation, the absorbance at 450 nm was measured using a microplate reader. The results are presented as mean ± SD (n = 4).

**5. Cell killing assessment**

The cell-killing efficiency was assessed in combination with near-infrared (NIR) laser irradiation. Briefly, primary MB49 tumor cells were obtained through enzymatic digestion and seeded into a 96-well plate at a density of 5×10^3^ cells per well in 100 μL of fresh DMEM medium containing 10% fetal bovine serum (FBS), 100 units/mL penicillin, and 100 μg/mL streptomycin. The cells were allowed to adhere and grow for 24 hours. In the second group, cells that had been exposed to different concentrations of cSiCe&ICG for 8 hours were subjected to irradiation using an 808 nm laser at a power density of 1.0 W/cm^2^ for 5 minutes. Both groups were then incubated for an additional 12 hours before the viability of the remaining live cells was determined using the MTT assay.

**6. Liver/dead cell determination**

Calcein-AM/PI staining experiments were performed to assess the in vitro anti-tumor activity and underlying mechanisms. MB49 tumor cells were seeded into a 6-well plate at a density of 1×10^5^ cells per well with 1 mL of fresh DMEM medium containing 10% fetal bovine serum (FBS), 100 units/mL penicillin, and 100 μg/mL streptomycin. The cells were cultured for 24 hours. Following this, the lung tumor cells were treated with different formulations: PBS, ICG+L, cSiCe&ICG, mSiCe&ICG+L, and cSiCe&ICG+L. The first three groups were exposed to the respective treatments for 24 hours. In the last group, after 8 hours of incubation, the cells were subjected to laser irradiation (1.0 W/cm^2^, 5 minutes) and then further incubated for an additional 12 hours. Finally, all cells from the four groups were stained with Calcein-AM/PI, and the live and dead cells were observed using CLSM.

**7. Cell apoptosis evaluation**

Cell apoptosis analysis was conducted in parallel. Cell samples were prepared following treatment with PBS, ICG+L, cSiCe&ICG, and cSiCe&ICG+L, respectively. The first three groups were exposed to the corresponding treatments for 24 hours. In the fourth group, cells were irradiated with a laser (1.0 W/cm^2^, 5 minutes) after 8 hours of incubation, followed by an additional 12 hours of culture. Subsequently, Annexin V/PI staining was carried out, and all cells from the four groups were harvested through enzymatic digestion. The extent of cell apoptosis was then quantified using flow cytometer.

**8. Measurement of blood circulation half-life**.

Chiral and nonchiral probes were first administered to mice via tail-vein injection, and blood samples were collected at predetermined time points. The nanoparticle probes in the blood were digested using a mixture of hydrogen peroxide and nitric acid, and the resulting samples were diluted in 5% nitric acid. The Ce concentration in the blood was then quantified by ICP-OES. An exponential decay curve was fitted by plotting the blood Ce concentration against time after injection. The Ce concentration in the injected rare-earth probes was also measured. Based on the fitted curve, the time required for the blood Ce concentration to decrease to half of its initial value was defined as the blood circulation half-life.

**Part 2: Supplementary Figures**

**Figure S1.** TEM images of cSiCe When Ce precursor (CeCl_3_•6H_2_O) was added at 0, 6, or 12 h after the addition of TEOS/cyclohexane.

**Figure S2.** SEM image of cSiCe.

**Figure S3.** (A) Specific surface area and (B) pore size distribution of cSiCe.

**Figure S4**. The calibration curve of ICG.

**Figure S5.** The percentage of ICG fluorescence intensity after incubated cSiCe&ICG with serum for various periods**.**

**Figure S6.** ICG and Ce ions released from cSiCe&ICG after incubated with pH6.5 buffer for various hours.

**Figure S7**. TEM image of mSiCe&ICG.

**Figure S8.** XRD spectra of cSiCe&ICG and mSiCe&ICG.

**Figure S9.** Size variations of cSiCe&ICG and mSiCe&ICG.

**Figure S10**. Zeta potential values of cSiCe&ICG and mSiCe&ICG.

**Figure S11**. Size variations of cSiCe and mSiCe after dispersed PBS for different periods.

**Figure S12.** TEM image of mSiCe&ICG after incubated with pH6.5 buffer for 36 h.

**Figure S13.** (A) Full XPS spectra of cSiCe&ICG. (B) High-resolute Ce 3d spectrum.

**Figure S14.** ESR spectra of cSiCe&ICG and mSiCe&ICG**.**

**Figure S15.** GSH consumption ability of cSiCe after weak acid treatment.

**Figure S16**. Mean fluorescence intensities in MB49 cells after various treatments for 8 hours.

**Figure S17.** (A) Flow-cytometer analysis and (B) corresponded mean fluorescence intensities of MB49 cells post-incubation with ICG, mSiCe&ICG and cSiCe&ICG.

**Figure S18.** CLSM of CT26 and 4T1 cells after treated with cSiCe&ICG or mSiCe&ICG, respectively.

**Figure S19.** Relative Ce ions in normal cells after incubated with cSiCe&ICG or mSiCe&ICG.

**Figure S20.** Internalization of (A, C) cSiCe&ICG and (B, D) mSiCe&ICG under treatment of various endocytosis inhibitors.

**Figure S21.** Mean fluorescence intensities from ROS in MB49 cells after various treatments for 8 hours.

**Figure S22.** Flow-cytometer analysis of the fluorescence intensity from DCFH-DA in the tumor cells after ICG, cSiCe&ICG, mSiCe&ICG+L and cSiCe&ICG+L.

**Figure S23.** Mean fluorescence intensities from •OH in MB49 cells after various treatments for 8 hours.

**Figure S24.** Red to green ratio of JC-1 in tumor cells after various treatments.

**Figure S25.** (A) Flow-cytometer analysis of MB49 cells stained by JC-1 following exposure to PBS, ICG+L, cSiCe&ICG, mSiCe&ICG+L, and cSiCe&ICG+L.

**Figure S26.** ICG fluorescence intensity in the central tumor region at 12 h after intravenous injection of mSiCe&ICG or cSiCe&ICG, respectively.

**Figure S27**. Quantitative measurements of signal intensities from ex vivo assessments of organs and tumor tissues in mice treated with cSiCe&ICG, mSiCe&ICG and ICG.

**Figure S28.** Mean bioluminescence intensities in bladder tumor-bearing mice at 30 days following surgery performed under either NIR-II fluorescence imaging guidance with cSiCe&ICG or conventional white light illumination.

**Figure S29**. H&E stained pictures of vital organs after administration of PBS+L, cSiCe+L, mSiCe&ICG+L and cSiCe&ICG+L for 21 days.

**Figure S30**. Partial blood count data indicators of BALB/c mice after intravenous injection of cSiCe&ICG for 1 and 2 weeks, respectively.

**Figure S31**. Partial blood biochemical indicators of BALB/c mice after intravenous injection of cSiCe&ICG for 1 and 2 weeks, respectively.

**Figure S32.** Residual percentage of (A) Ce ions and (B) ions in the reticuloendothelial system after injection of cSiCe&ICG for 7, 14 and 28 days.

**Figure S33.** Serum levels of IL-10, TNF-α, and IFN-γ in tumor bearing mice after 3 days post-treatments of cSiCe&ICG, mSiCe&ICG and ICG.
